# Reducing anxiety and enhancing innovation in nurses: a psychological capital intervention study in China

**DOI:** 10.1186/s12912-025-02838-y

**Published:** 2025-02-22

**Authors:** Dexiu Yan, Lan Chen, Mengyao Li, Yuanyuan Zhang, Yaqing Zhang

**Affiliations:** 1 Department of Nursing, Shanghai General Hospital, Shanghai, China; 2 Shanghai Jiao Tong University School of Nursing, 227 South Chongqing Road Huangpu District, Shanghai, China; 3 School of Nursing, LKS Faculty of Medicine, The University of Hong Kong, Hong Kong, China

**Keywords:** Psychological capital, Anxiety, Innovation, Intervention

## Abstract

**Aim:**

This study aimed to test whether increasing psychological capital through psychological capital interventions affects anxiety and innovation among nursing staff through an experimental study.

**Method:**

This study utilized a two-group (experimental and wait-list control) pre-test and post-test design. In August 2022, eighty clinical nurses from the same hospital were randomly assigned to either the experimental group (40) or the wait-list control group (40) to complete self-report questionnaires at pre-intervention (T1), post-intervention (T2), and one month post-intervention (T3). The experimental group received a 6-week training on the psychological capital intervention.

**Result:**

The scores of anxiety, psychological capital, and innovative behaviour of experimental subjects in the two groups before and after the intervention showed no significant difference in between-group effects (P > 0.05). In contrast, the time effect and between-group × time effect were significant (P < 0.05), and the trends of anxiety, psychological capital and innovative behaviour over time differed between the two groups of nurses.

**Conclusion:**

Online psychological capital interventions can weaken the constraints of time and space to maximize the effective development of nursing staff's psychological capital, reduce anxiety, and improve nurses' innovative behaviors.

## Introduction

Nurses are in a challenging work environment. Most nurses are dealing with a population of patients who are seriously ill [1]. In order to effectively save the health of patients, most nurses are in a constant state of high pressure and need more innovation to keep up with the pace of medical development [2]. Stress impairs mental health and leads to many psychosomatic disorders such as anxiety, high blood pressure, insomnia, and weakened immune system [3].A study showed that at least 23.2% of healthcare workers have anxiety [4]. How to effectively reduce anxiety among nurses is also a considerable challenge for nurse managers [5].

Similarly, the stress in nursing care not only from patients but also from the rapid advances in medical technology [6]. In particular, the role of nurses has become increasingly prominent as the medical community recognizes the importance of patient care. As a result, the importance of innovation in nursing is becoming increasingly evident [7]. The American Nurses Association(ANA) [8] defines innovation as putting new ideas into practice or putting existing ideas into practice in new ways, and it points out that addressing the complexity of healthcare services requires innovative solutions and creative approaches to the status quo. Effective implementation of clinical innovation can help nurses perform better clinical work, reduce work stress, and, in turn, alleviate the anxiety condition of nursing staff.

Literature on anxiety relief and enhancement of innovative behavior has shown that personal psychological capital plays an important role in nurses’ anxiety and innovative behavior [9, 10]. Based on the Conservation of Resources Theory (COR) [11], we sought to investigate the effect of psychological capital (PsyCap) on anxiety and innovative behavior of clinical nursing staff. Recognizing the development of positive psychology and its potential, Luthans et al. [12] conceptualized the PsyCap construct in a work setting. PsyCap is defined as "a state of positive psychological development in an individual characterized by i: Confidence (self-efficacy) to take on and put forth the effort necessary to succeed in challenging tasks. ii: Positive attributions for present and future success (Optimism). iii: Adherence to goals and redirecting the path of goals when necessary to achieve success (Hope). And iv: Sustaining and bouncing back or even surpassing to achieve success when plagued by problems and adversity (Resilience) [12]. When a person has the psychological resources (self-efficacy, optimism, hope, and resilience), they are said to be PsyCap.

The PsyCap intervention (PCI) is a two-hour intervention [13]. The delivery of the PCI training program is based on active participation and adult learning. Luthans et al. [13] argued that the PCI program is advantageous because it is short and minimises work schedule interruptions. Additionally, this short training is consistent with a short-term goal identification program.The PCI training includes discussions, reflection exercises, and writing exercises. The development of each component of PsyCap is based on sound theoretical principles. For example, hope is developed through multiple pathway planning [14]. Self-efficacy is developed through task mastery, alternative learning (observing experts), and social persuasion [15]. Resilience is developed through increased awareness of participants' assets [16]. Optimism is developed through the principles of positive expectations and positive attributions [17]. PCI is based on the positive psychology literature (the principle of "intentional activity"). Lyubomirsky et al. [18], a prominent positive psychology scholar, suggests that 40% of positivity is developed through "conscious activity" development. This positive intentional activity helps to foster positive emotions and develop positive cognitions and behaviours [19].

Many studies have focused on the factors that influence PsyCap and the outcomes it leads to [20], but have not examined PsyCap interventions. Luthans et al. [12] proposed the intervention model to operationalize and implement PsyCap interventions. Previous PsyCap intervention studies have several commonalities. First, they are based mainly on the PCI model. Second, they provide one to three hours of intensive training. Third, they focused primarily on the effectiveness of the PsyCap intervention but neglected its impact on other work-related outcomes, with only one study testing the impact of the intervention on on-the-job performance [21]. Fourth, the participants were all from Western countries. Fifth, most of the main population it intervened with were employees of companies.

However, due to the unique demands and flexible nature of nursing work schedules, implementing a centralized training approach in the workplace poses significant challenges, particularly in China. Nursing staff often have varying shifts and responsibilities, making it difficult to identify a universally suitable time for training sessions. Moreover, urgent or unpredictable work duties may prevent nurses from participating in interventions as scheduled, further complicating centralized implementation.To address these challenges, this study aimed to adapt and expand the application of the PsyCap intervention to better align with the cultural and professional context of mainland China, focusing on both theoretical and practical enhancements. Firstly, the intervention incorporated innovative objectives designed to complement and enrich the existing Psychological Capital Intervention (PCI) model, tailoring it to the unique needs of the nursing profession. Secondly, the study shifted from a traditional in-person training approach to a flexible, daily online self-study format, allowing nurses to engage with the intervention at their convenience, regardless of their work schedules.Furthermore, grounded in the Conservation of Resources (COR) theory, this study sought to explore the broader impact of the intervention on both PsyCap and other critical work-related outcomes. In particular, the study examined the intervention's effects on anxiety levels and innovative behavior, which are vital components of professional resilience and adaptability in the demanding field of nursing. By addressing these aspects, the study aimed to provide a more practical and accessible approach to PsyCap development while contributing to a deeper understanding of its implications in the healthcare sector.

## Materials and methods

### Study design

This study utilized a two-group matched pretest, post-test, and follow-up test design utilizing a quasi-experimental study. In clinical studies with human subjects, even if a randomization strategy is adopted for grouping, it is still difficult to achieve idealized randomized grouping due to the constraints of clinical practice or ethical norms. This study was approved by Chinese Clinical Trial Registry (Clinical trial number: ChiCTR2100045389, 2021–04–14).

### Sample and setting

Participants were clinical nurses from a tertiary care hospital in Shanghai, China. PASS 15.0 was used for sample size calculation. Based on previous studies [22], the test efficacy 1-βwas set at 0.8, and the two-sided test criterionɑ = 0.05 was taken to calculate that the sample sizes of the experimental group and the waiting-list control group should be 32 cases each. Considering that sample loss was likely to occur in the intervention study, the final number of experimental group and waiting-list control group included in this study were 40 people each.

Inclusion criteria: i: Obtained the license to practice nursing. ii: Engaged in clinical work for more than one year. iii: Bachelor's degree or above. iv: Voluntary participation in this study.

Exclusion criteria: i: Further training nurses. ii: The intervention mainly concerned the daily work of frontline nurses, thus excluding those not involved in clinical work (auxiliary, administrative and logistical departments). iii: Previously trained in other psychological capital intervention programs. iv: Failed to participate in the whole process of this intervention study.

### Sampling and recruitment methods

In July 2022, we recruited study participants by putting up posters and posting notices in a hospital. In order to minimize the disruptions created by daily training and the hospital atmosphere, the subjects of this study were recruited in a single hospital. The posters and notices included the research purpose of this training, the significance of the study, what the participants would learn, the rights and obligations of the participants (the participants could withdraw from this study at any time), and the compensation and incentives that the participants would receive (a certificate of training for one course).. After one month, we recruited a total of 80 nurses and used a simple randomization method whereby one non-participant randomly numbered the study participants and another non-participant used Excel software to generate a random sequence table based on which the 80 were randomly divided into the experimental and control groups.

### Intervention

The waiting-list control group received regular training in the hospital, including nursing room visits, operation training, nosocomial infection training, business study, management training, etc. The intervention program lasted 6 weeks (The intervention focuses on the four themes of psychological capital as well as an introduction and summary), with each module lasting one week. It is mainly conducted in video sessions, which last for about 30–40 min/day(See supplement files). The intervention program was loaded into the Learning Link software(Xue Xi Tong), and each study participant had a separate personal account. The interventionist was a graduate student. During the intervention process, the interventionist would send a standardized practice reminder message to the participants of the experimental group every week, "Students, the first chapter of the psychological capital training, "Understanding Psychological Capital" has been opened, please watch it in time, and do not forget to test the chapter and discuss it ~ ". Changes in psychological capital, innovative behavior and related variables were assessed in the intervention and waiting-list control group before (T0) immediately after the end of the intervention (T1) and one month after the intervention (T2), and the innovative outputs of the study participants were collected six months after the intervention.

### Measurement

#### Demographic information

General demographic information was collected, including age, length of service, job title, education level, position assumed, and marital status.

#### Nurses' innovative behavior scale

The Nurses' Innovative Behavior Scale developed by Bao et al. [23] was used to measure the frequency of nurses in carrying out innovative activities at work. It contains three dimensions with ten entries, namely: generating ideas (3 entries), getting support (4 entries) and realizing ideas (3 entries). The scale was scored on a 5-point Likert scale with higher scores, with higher scores indicating more innovative behaviors on the job The Cronbach's alpha coefficient of the source scale was 0.879, and the coefficients of the three dimensions ranged from 0.746 to 0.870. In this study, the Cronbach's alpha coefficient for the scale was 0.912, and the coefficients for the dimensions were 0.835, 0.809, and 0.902.

#### Psychological capital scale for nurses

The psychological capital scale for nurses, originally developed by Luthans et al. [13] (2006) and revised by Chinese scholar Luo Hong et al. [24], was utilized to assess nurses' psychological capital. This scale comprises four dimensions with a total of 20 items: self-efficacy (6 items), hope (6 items), resilience (5 items), and optimism (3 items). Responses were measured using a 6-point Likert scale, with higher scores indicating better psychological capital. The original scale demonstrated strong reliability, with Cronbach's alpha values ranging from 0.89 to 0.98.

#### Generalized Anxiety Scale (GAD-7)

The GAD-7 (7-items Generalized Anxiety Disorder Scale) was developed by Spitzer's [25] team in 2006 using a large sample of data from primary care visits.The Chinese version of the GAD-7 scale was Chineseized by He et al. [26] in 2010, and the Cronbach's alpha coefficient of the Chinese version of the GAD-7 was 0.898, and the retest reliability coefficient was 0.856. The GAD-7 asks patients about changes in their mental mood in the last two weeks, and the main components include "feeling tense, anxious, or angry" and "cannot stop or cannot control". "Worrying uncontrollably or uncontrollably," "Worrying excessively about many things," "Difficulty relaxing," "Difficulty sitting still," "Easily irritated or provoked," "Feeling scared, as if something terrible is going to happen." According to the above symptoms, "not at all", "sometimes", "most of the time", and "almost every day" scores were 0–3, and the score range was 0–21. Higher scores indicate greater anxiety in participants. GAD-7 can not only be used as a screening test for GAD but also can be used to assess the severity of the condition based on the scores, and according to the scores of 5, 10, and 15, it was rated as a mild, moderate, or severe anxiety disorder.

### Statistical analysis

SPSS 23.0 was used for data analysis. Descriptive analyses described demographic data, normal distribution, GAD-7, creative behavior, and psychological capital scores collected at T0, T1, and T2. Repeated measures ANOVA was used to compare GAD-7, innovative behavior, and mental capital scores between the two groups, and statistical significance was established at a p < 0.05. Generalized estimating equations (GEE) were used to analyze the composition and time variation of the variables to solve the problem of non-independence of repeated measures data to obtain robust parameters.It is possible to explore the main effect of the independent variable on the dependent variable and also to detect interactions between different independent variables.

### Ethical considerations

The study was approved by the Ethics Committee of Shanghai Jiaotong University(SJUPN-202121), which complied with the Declaration of Helsinki. All participants were informed of the study’s objectives, procedures, and potential risks and were informed of their right to withdraw at any time. Written consent was obtained from the participants.

## Results

### Characteristics of the participants

The mean age of the participants was 28.25 ± 0.685. Table 1 showed no significant difference (*p* > 0.05) between the two groups of study participants regarding age and demographic information such as years of working experience and assumed positions. Table 2 showed no significant difference (*p* > 0.05) between the two groups of study variable.

**Table 1:** General information of the study population

Item	Total sample (*N* = 80)	Experimental group (*n* = 40)	Waiting-list control group (*n* = 40)	*t*/*x*^2^/Z	*P*
Age	28.25 ± 0.685	29.05 ± 3.796	27.45 ± 7.053	−1.263	0.210
Length	6.90 ± 3.008	7.00 ± 3.088	6.80 ± 3.123	−0.228	0.774
Weekly Night Shift	1.39 ± 0.665	1.40 ± 0.698	1.38 ± 0.638	−0.194	0.847
Overtime hours per month	1.60 ± 0.789	1.60 ± 0.810	1.60 ± 0.778	0.000	1.000
Position^a^					
Clinical nurse	79 (98.8)	39 (97.5)	40 (100)	1.013	0.314
Lead Teacher	1 (1.3)	1 (2.5)	0 (0)		
Marriage					
Single	42 (52.5)	20 (50)	22 (55)	0.201	0.654
Married	38 (47.5)	20 (50)	18 (45)		

^a^is Fisher's exact test

**Table 2:** Analysis of baseline innovative behavior and influencing factors of the two groups of study participants

Variable	Waiting-list control group (*n* = 40)	Experimental group (*n *= 40)	t/**χ2**	p
**Innovation Behavior**	3.32 ± 0.75	3.13 ± 0.70	0.102	0.274
Generating ideas	3.73 ± 0.61	3.68 ± 0.59	0.311	0.756
Getting support	3.17 ± 0.77	2.95 ± 0.77	1.268	0.209
Realizing ideas	2.84 ± 0.98	3.10 ± 1.04	1.142	0.257
**Psychological Capital**	4.57 ± 0.68	4.47 ± 0.74	0.227	0.821
Self-efficacy	4.54 ± 0.80	4.53 ± 0.81	0.047	0.962
Hope	4.49 ± 0.69	4.38 ± 0.84	0.656	0.514
Optimism	4.70 ± 0.85	4.46 ± 0.91	1.268	0.209
Resilience	4.63 ± 0.73	4.53 ± 0.77	0.593	0.555
**Anxiety**	4.23 ± 3.09	4.68 ± 4.06	0.557	0.579
Mild Anxiety	37 (92.5%)	36 (90%)	0.157	0.692
Moderate Anxiety	3 (7.5%)	4 (10%)		

**Fig. 1:**
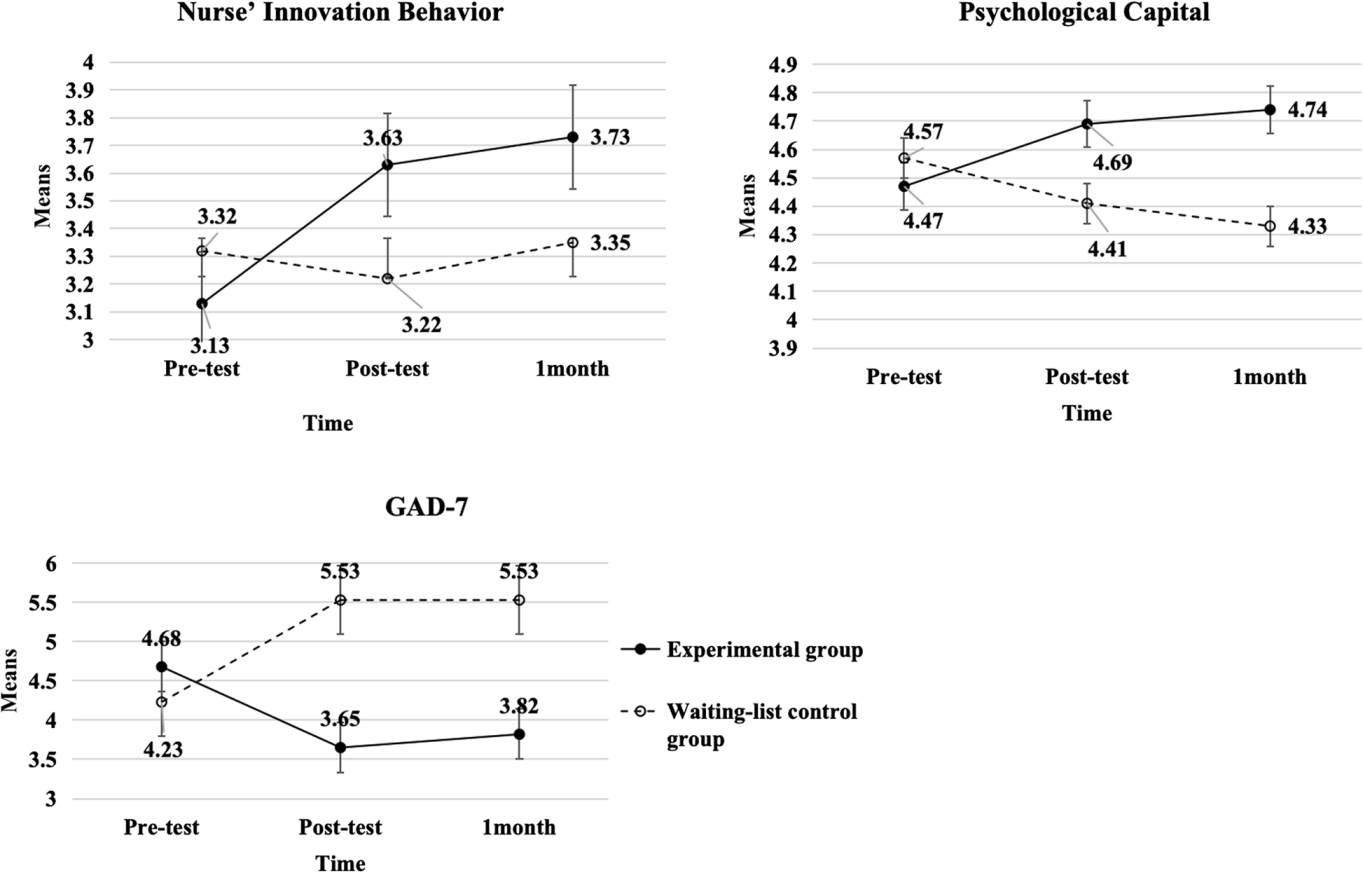
The curves of changes in psychological capital, innovative behavior, and GAD-7 of participant

The length of video viewing for the 40 study participants was 177.69 ± 16.39 min (0 to 170), and the number of discussion responses was 3.15 ± 1.86 times (0 to 5). There was no statistically significant difference between the mean innovative behavior scores and mean psychological capital scores of the experimental group and the waiting list control group (Table 2). At the three measurement time points, the mean values of innovative behaviour and psychological capital gradually increased, and the mean value of GAD-7 gradually decreased in the experimental group, indicating a gradual increase in innovative behavior and psychological capital and a gradual decrease in anxiety in this group. In contrast, the waiting-list control group showed the opposite results. The changes in the measured values of the two groups at the three-time points are shown in Fig. 1.

### Effectiveness of psychological capital interventions

Table 3 showed the effectiveness of the psychological capital intervention in alleviating anxiety and enhancing the psychological capital and innovative behavior of clinical nurses. In the generalized estimating equation analysis results, when comparing the experimental and waiting-list control group, the intervention and waiting-list control group did not show significance in the psychological capital, anxiety, and innovative behavior scores. Regarding the time effects, participants showed significant changes over time in psychological capital, anxiety in Post-test 2, and innovative behavior in Post-tests 1 and 2, as compared to the pretest. Regarding the group and time interaction effects, it was found that the experimental group showed statistically significant differences in both psychological capital and innovative behaviour in both post-test 1 and 2 when compared to the waiting-list control group. Anxiety showed a statistically significant difference in post-test 2.

**Table 3:** GEE analysis of the effects of psychological capital intervention

Variable	Psychological capital				Anxiety				Innovative behavior			
	β	SE	**Wald χ2**	P	β	SE	**Wald χ2**	P	β	SE	**Wald χ2**	P
Intercept (Experimental group vs Wating-list)^a^	2.919	0.204	204.976	** < 0.001**	−1.225	0.365	11.284	**0.001**	1.563	0.1947	64.424	< 0.001
Group (Experimental group vs Wating-list)^a^	−0.059	1.174	0.003	0.960	0.175	0.516	0.115	0.734	1.442	1.141	1.597	**0.206**
Time^b^												
T0vsT1	0.289	0.158	3.335	0.068	0.825	0.516	2.559	0.110	0.435	0.1689	6.635	**0.002**
T0vsT2	0.429	0.143	9.032	**0.003**	1.100	0.516	4.549	**0.033**	0.650	0.1617	16.157	< 0.001
Interaction^c^												
Experimental group × post-test 1^d^	−0.540	0.241	5.005	**0.025**	−1.225	0.729	2.821	0.093	−0.490	0.2425	4.083	**0.043**
Experimental group × post-test 2^e^	−0.919	0.259	12.612	** < 0.001**	−1.500	0.729	4.230	**0.040**	−0.767	0.2655	8.359	**0.004**

^a^Reference: Waiting-list control group

^b^Reference: pretest

^c^Reference: Waiting-list control group x pretest

^d^Experimental group (post-test 1—pretest)—Waiting-list control group (post-test 1—pretest)

^e^Experimental group (post-test 2—pretest)—Waiting-list control group (post-test 2—pretest)

## Discussion

This study aimed to examine the impact of a psychological capital intervention on improving clinical nurses' psychological anxiety and enhancing psychological capital and innovative behaviors. Since Luthans proposed the psychological capital intervention model in 2006 [13], many scholars have explored its effects on positive and negative psychological factors. Many studies have explored the role of psychological capital interventions in improving psychological conditions in research. For example, Patnaik et al. [27] among telecom employees in India. Song et al. [28] among depressed patients in China and Liang et al. [29] among left-behind children in China. The results of the present study were similar to the above studies in that the experimental group reported significantly higher levels of PsyCap after PCI compared to pre-intervention. However, the waiting-list control group did not report any significant increase in PsyCap levels after the intervention. Similarly, participants in the treatment group had significantly lower anxiety significantly higher innovative behavior scores. However, the waiting-list control group did not report any significant changes in anxiety or innovative behavior scores. Thus, these findings suggest that an increase in PsyCap over time helped participants reduce anxiety and elevate levels of psychological capital.

Specifically, PsyCap may focused more on difficulties at work and helping people deal with or overcome these difficulties [30], which may be caregivers’ leading cause of anxiety. Caregivers tend to view problems from a negative perspective, caregivers are susceptible to long-term exposure to patients suffering from illness [31], and PCI can help them adjust their emotions and improve their work [32]. Given its potential to reduce anxiety and improve emotional adjustment, interventions that aim to enhance PsyCap may be a valuable tool for caregiver support programs. Training caregivers to strengthen their PsyCap through targeted interventions, such as resilience training, hope-building exercises, and self-efficacy-enhancing activities, can foster long-term psychological well-being and reduce the risk of caregiver burnout. Research has shown that PsyCap interventions can lead to tangible improvements in both psychological outcomes and job satisfaction, suggesting that such approaches can also help caregivers feel more competent and less stressed in their roles [32]. Given these academic insights, PsyCap is a positive psychological resource that can serve as one of the critical psychological resources needed to cope and positively assess situations in challenging situations successfully [33, 34]. Thus, PsyCap may help reduce anxiety [35].

In addition, the literature on innovative behavior suggests that while innovative behavior is objective and external, it is also external to subjectivity in nature [38]. Subjective factors are likely to be positively influenced by the positive attributes of PsyCap individuals. Drawing on Hobfoll 's [27] COR Theory, people with a high PsyCap were likely to show more innovation because they believe they had positive psychological resources to cope with adversity [39]. Therefore, PsyCap can assist to enhance innovative behavior. Drawing from the research of Yan et al. [9] and integrating COR theory with PsyCap, a causal relationship between PsyCap, anxiety, and innovative behavior emerges. According to this view, PsyCap can reduce anxiety by providing individuals with the necessary psychological resources to cope with stressors effectively. The reduction in anxiety enables individuals to focus more on creative problem-solving and innovation, as they are less preoccupied with negative emotions or the fear of failure.

Although this study was conducted online, participants demonstrated good compliance [36]. Compared to traditional education, online education has the advantages of high satisfaction, efficient learning, and more autonomous learning [37]. The intervention form of online training can maximize the time and space constraints, and the study participants can complete the video task point learning according to their personal free time [38], which does not increase the burden of clinical nurses. Although the psychological capital intervention cannot change the nursing work itself, it can help the nurses to sort out the existing available resources and help them to control their work better so that they are willing to look at work and life with a more optimistic mindset and alleviate anxiety. According to the social exchange theory [39], the more internal resources an individual has at his or her disposal will correspondingly promote the individual's sense of control at work so that the individual will be more willing to produce external outputs and perform behaviors that benefit the organization, and innovative behaviors will increase.

About the limitations of this study. The research sample of this study is limited to one organization, the sample size of this study is small, and the results cannot be generalized. Therefore, we recommend designing further studies with larger sample sizes in China. In addition, our use of self-report methods to measure all variables may lead to common method bias. Additionally, this study did not collect changes in indicators after three months of nurse intervention as nurses were overwhelmed with the workload caused by the disease as a result of the three-month defense that began in Shanghai due to the novel coronavirus. Future studies of PsyCap interventions should consider examining the effect of duration through longer follow-up.

## Conclusion

The development and application of psychological capital (PsyCap) interventions have seen considerable evolution since their inception in 2006 [14]. Initially focused on corporate employees and students, the scope of PsyCap interventions has expanded to a broader range of sectors. However, there is still limited exploration of how these interventions can be effectively applied in healthcare settings, particularly among nurses and other healthcare workers. Our study highlights the beneficial effects of psychological capital interventions for caregivers. Specifically, such interventions have been shown to alleviate anxiety, enhance psychological capital (comprising hope, efficacy, resilience, and optimism), and improve innovative behavior. Online PsyCap interventions empower nurses by fostering a positive mindset that can better equip them to manage stress, cope with challenges, and engage in creative problem-solving. This leads to improved job performance, better patient care, and the cultivation of a more resilient healthcare workforce. Psychological capital interventions offer a promising solution to reduce anxiety, enhance resilience, and foster innovative behaviors among nurses. Nursing managers play a crucial role in embedding these interventions into their practice, promoting a culture of psychological health, and ensuring that nurses are not only equipped to handle the emotional demands of their work but are also empowered to contribute to the ongoing innovation in healthcare delivery.

## Data Availability

Due to the sensitive nature of the questions asked in this study, survey respondents were assured raw data would remain confidential and would not be shared.
